# Chromosome instability in neuroblastoma: A pathway to aggressive disease

**DOI:** 10.3389/fonc.2022.988972

**Published:** 2022-10-20

**Authors:** Lucia Paolini, Sajjad Hussain, Paul J. Galardy

**Affiliations:** ^1^ Department of Pediatrics, University of Milano-Bicocca, San Gerardo Hospital, Monza, MI, Italy; ^2^ Department of Pediatric and Adolescent Medicine, Mayo Clinic, Rochester, MN, United States; ^3^ Division of Pediatric Hematology-Oncology, Mayo Clinic, Rochester, MN, United States

**Keywords:** neuroblastoma, chromosome instability (CIN), aneuploidy, deubuquitylases, mitosis, chromosome missegregation, mitotic spindle

## Abstract

For over 100-years, genomic instability has been investigated as a central player in the pathogenesis of human cancer. Conceptually, genomic instability includes an array of alterations from small deletions/insertions to whole chromosome alterations, referred to as chromosome instability. Chromosome instability has a paradoxical impact in cancer. In most instances, the introduction of chromosome instability has a negative impact on cellular fitness whereas in cancer it is usually associated with a worse prognosis. One exception is the case of neuroblastoma, the most common solid tumor outside of the brain in children. Neuroblastoma tumors have two distinct patterns of genome instability: *whole-*chromosome aneuploidy, which is associated with a better prognosis, or segmental chromosomal alterations, which is a potent negative prognostic factor. Through a computational screen, we found that low levels of the de- ubiquitinating enzyme *USP24* have a highly significant negative impact on survival in neuroblastoma. At the molecular level, USP24 loss leads to destabilization of the microtubule assembly factor CRMP2 - producing mitotic errors and leading to chromosome missegregation and whole-chromosome aneuploidy. This apparent paradox may be reconciled through a model in which whole chromosome aneuploidy leads to the subsequent development of segmental chromosome alterations. Here we review the mechanisms behind chromosome instability and the evidence for the progressive development of segmental alterations from existing numerical aneuploidy in support of a multi-step model of neuroblastoma progression.

## Introduction

Advanced sequencing technology has produced an explosion of information regarding cancer genomes. Such efforts have generated valuable information regarding signaling networks and new therapeutic entry points. The majority of cancers, however, are characterized by variations in chromosome numbers (aneuploidy) or copy number alterations that affect small to large segments of chromosome arms (1). These relatively large genomic alterations affect the expression of many genes and perhaps understandably have a correspondingly less linear mechanistic link to malignant behavior. Aneuploidy is associated with poor outcomes in many cancer subtypes including prostate (2), breast (3–5), lung (6), and others (7). In neuroblastoma, whole chromosome aneuploidy is associated with a good prognosis, whereas segmental chromosome alterations associated with poor outcomes – even when co-existing with whole chromosome aneuploidy (8). It is notable that the type of genomic alteration (gains or losses of large chromosome segments) has a greater impact on tumor behavior than which specific chromosome region is affected. This leads us to speculate that it is the underlying mechanism that produces these alterations may itself that is the prognosis-driving event. Here we review the mechanisms that underlie whole chromosome aneuploidy as well as the mechanistic link between aneuploidy and the subsequent development of segmental chromosome alterations. We hypothesize that tumors harboring segmental alterations may have developed through a multi-step process that begins with mitotic missegregation of whole chromosomes and proceeds to segmental alterations.

## Genomic instability and cancer

The term genomic instability indicates a cells tendency to accumulate new genomic alterations (3, 9). This broad term includes defects ranging from small insertions/deletion to large segmental and whole chromosomal alterations. Whole chromosome instability (CIN) refers to a state in which there is an increased incidence of cell divisions resulting in the loss of gain of whole chromosomes (10, 11). This is to be distinguished from aneuploidy – which refers to a cell state of possessing too many or few chromosomes. Otherwise normal cells occasionally undergo chromosome segregation errors in mitosis – albeit at a low rate. Frequently, the resulting daughter cells undergo apoptotic death and therefore do not lead to aneuploidy. Consequently, there is a low (but non-zero) rate of aneuploidy in otherwise normal tissues. Increased aneuploidy therefore requires there to be an increased rate of chromosome segregation errors (the state of CIN) or that there be an increased likelihood of cell survival after mitotic errors that occurred at the otherwise normal low frequency. Differently stated, aneuploid cells may result from CIN – but they do not necessarily exhibit ongoing CIN. It has been estimated that up 80% of cancer cells have abnormalities related to chromosome missegregation (12, 13). Whole chromosome instability can be induced by multiple mechanisms during cell division, in particular errors during mitosis such as: centrosome replications errors, alterations of spindle assembly checkpoint, defect in sister chromatid cohesion, damage of microtubule attachments to chromosomes (7, 14). Furthermore, anti-neoplastic therapies can be a cause of chromosome segregation errors as well (15). Many – if not most – animal models with a predisposition to CIN due to engineered alterations in mitotic regulators have an increased tumor incidence, providing strong evidence for the *in vivo* impact of CIN on cancer development (16, 17).

Chromosomal instability and aneuploidy are generally associated with poor prognosis in solid cancers (1, 18, 19). Gene expression signatures reflecting CIN have been applied across many cancer types. While work from yeast and mammalian systems suggests that there are hundreds of genes that may cause – or protect cells from CIN, it is noteworthy that the over-expression of some classical oncogenes [e.g. KRAS; (20)] and loss of some tumor suppressors [e.g. PTEN, (21)] also leads to CIN. In most instances, tumors that exhibit a high CIN gene expression signature have worse prognosis (18, 22). In an analysis of 2125 patients, those with a higher than the median CIN70 score had a worse outcome (18). Similarly, the analysis of TCGA (*The Cancer Genome Atlas;* https://www.cancer.gov/about-nci/organization/ccg/research/structural-genomics/tcga) cohorts on several cancer types showed a tight inverse association between cancer cell aneuploidy and cancer-free and overall survival, not only in primary tumors but also those with metastases (19). These studies lead to the general conclusion that CIN is a cause – or marker – of aggressive cancer.

## Causes of CIN in cancer

The emergence of aneuploid cells depends on multiple pathways such as 1) mis-segregation of a chromosome(s) during mitosis resulting in daughter cells with non-modal chromosome numbers and 2) survival of the aneuploid cell. Increased numbers of aneuploid cells then may result from an increase in chromosome mis-segregation events (CIN), increased survival of aneuploidy progeny, or both (23). In this review, we will focus on the origins of CIN and the potential contribution of these mechanisms to NBL pathogenesis. However, we must recognize that events that interfere with cell death pathways – such as the loss of functional TP53 or other p53 superfamily genes, or gain of anti-apoptotic proteins such as BCL2, may also play a role in the development of CIN in NBL. Indeed, genes such as *CCNB1, PRC1, TPX2, AIRKB, NEK2* that are known regulators of cell cycle and accurate mitosis are over-expressed in high risk NBL tumors (22, 24).

**Figure 1:**
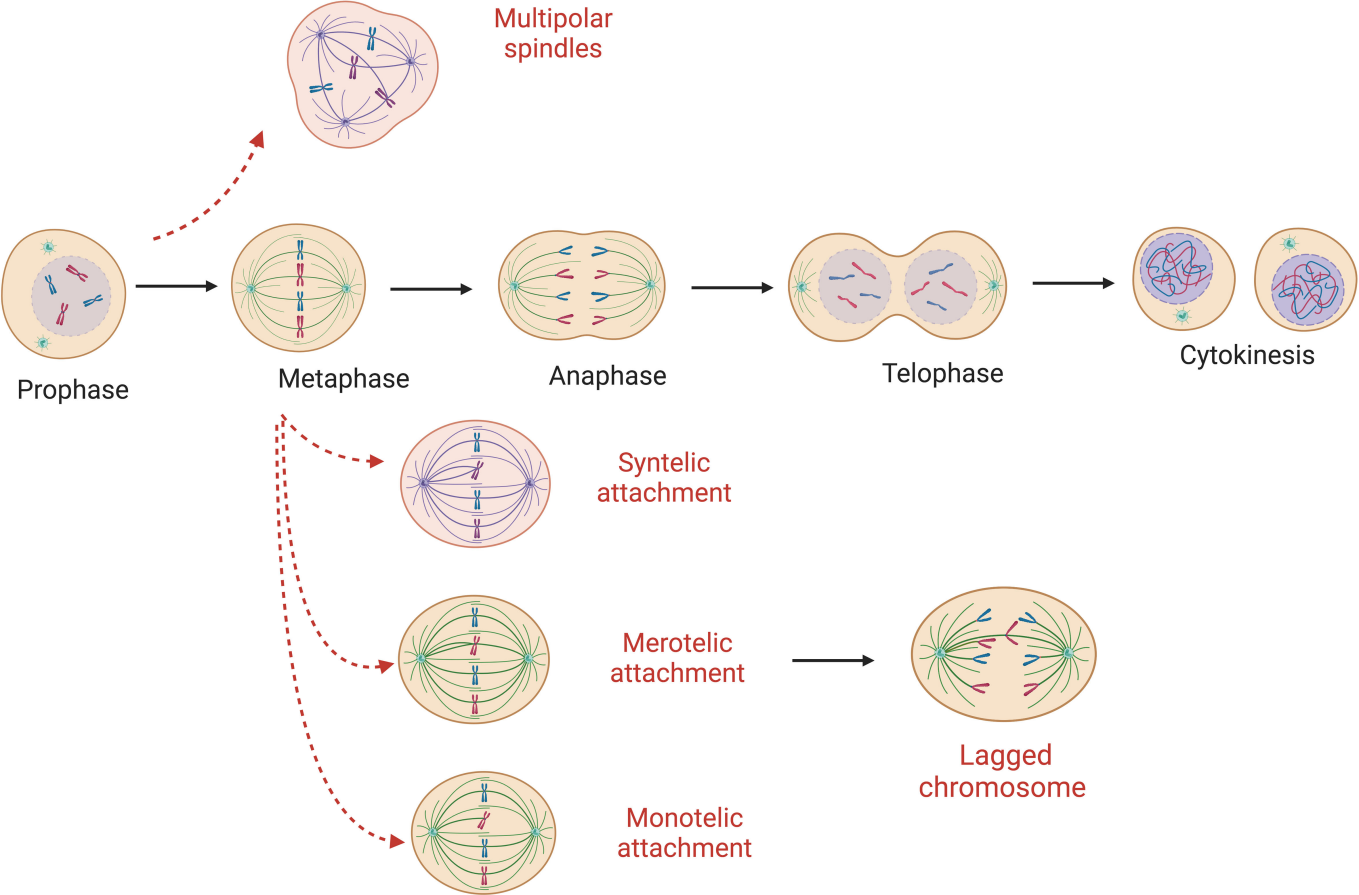
Abnormal microtubule attachments during mitosis. Red-dashed arrows show possible errors that can occur during cell replication ultimately leading to mis-segregated chromosomes. Here monotelic, syntelic, merotelic and multipolar spindle are represented. Absence of attachment and premature loss of sister chromatid cohesion are possible origins of W-CIN as well.

Aberrant attachments of spindle microtubules to the kinetochores of sister chromatids (**Figure 1**) is the most common cause of chromosome segregation errors in mitosis (25). In an error-free metaphase, each sister chromatid is attached to microtubules originating from only one spindle pole. There are different forms of incorrect attachment of the microtubules to the kinetochores such as: absence of attachment, monotelic, syntelic, and merotelic attachment, multipolar spindle and premature loss of sister chromatid cohesion. In the monotelic attachment only one chromatid sister has the kinetochore attached to the microtubules. This error is that which is most readily corrected – as the presence of an unbound kinetochore triggers the spindle assembly checkpoint (SAC) to delay anaphase onset. In other cases – such as syntelic (both sister chromatids linked to one spindle pole) and merotelic attachments (one chromatid bound to microtubules from more than one spindle pole) – it is possible for errors to be unrecognized by the SAC and therefore persist through mitosis (13). However, these errors result in a lack of tension that normally exists when paired kinetochores are pulled towards opposing spindle poles. This lack of tension is an activation signal for the aurora B error correction machinery – that leads to detachment – and reattachment of microtubule-connections until errors are corrected (26). Merotelic attachments are some of the more frequent errors in mitosis. They are particularly dangerous as they are ‘invisible’ to the SAC due to the presence of microtubule-kinetochore attachments (13). The origins of merotelic attachments may originate due to supernumerary centrosomes, defective centrosome separation, premature anaphase due to SAC errors, or defects within the aurora B correction process (27–29).

## CIN, aneuploidy, and neuroblastoma

Neuroblastoma (NBL) is a pediatric solid tumor originating from the sympathetic nervous system (30) that represents 7-8% of pediatric malignancies (31). It can originate from any sympathetic ganglia such as in the adrenal medulla (the most common site involved), the chest and neck (along the sympathetic nervous system chain), or the pelvis (para- aortic body; organ of Zuckerkandl) (31–33). According to the International Neuroblastoma Risk Group (INRG) Staging System (INRGSS), NBL is divided into locoregional disease (L1 and L2 determined respectively by the absence or presence of imaging defined risk-factors), or stage M in the presence of metastatic disease (34). Common locations of metastatic spread include the bone marrow, bone, lymph nodes, and liver. Lastly, is stage MS (metastatic special) described as a tumor with metastatic lesions of the skin, liver and bone marrow in patients aged from 0 to 18 months (35). What makes this ‘special’ is the tendency of stage MS tumor to undergo spontaneous resolution.

NBL is characterized by a wide molecular and clinical diversity (31, 36–39). Prognosis depends not only by the conventional risk markers such as age, stage and histopathology (40), but also on gene expression (41–43) and genomic signature of the tumor (31, 40, 44). CGH array studies have furthermore helped to classify neuroblastoma tumors in different groups depending on their genomic alteration patterns (37, 45, 46). Neuroblastoma exhibit recurrent genomic alterations that may be characterized as numerical (whole chromosome aneuploidy), or segmental. Common segmental chromosomal alterations include deletion of 1p (47); 3p; 11q (36, 48, 49); 4p; 9p and 14q chromosomes and gain of 1q; 2p and 17q (40, 50–54). In contrast to the frequent macro-genomic alterations, NBL has a generally low mutation burden, and few recurrently altered genes (55, 56). The two of best studied gene alterations are: *MYCN* (57), which is amplified from 20% to 25% of NBL and is correlated with a poor prognosis (58–60), and *ALK* mutations occurring in 6-9% of sporadic tumors. Germline mutation in *ALK* is also a major cause of familial neuroblastoma (61).

Numerical and structural alterations are correlated with specific clinical features and prognosis. Younger patients, usually less than 1 year old (38), are found to havetumors that exhibit mostly numerical genomic alterations (62), and are associated with localized stages of the disease and excellent survival rates. In contrast, patients with tumors harboring structural chromosome aberrations are usually older, present with advanced stage disease (52), and have tumors with more aggressive growth patterns (63, 64). In large study comparing various genomic patterns, Janoueix- Loresey and colleagues analyzed 493 tumors using array CGH with accompanying outcomes data (8). The 4-year progression free survival was over 90% in those tumors with only numerical alterations, compared with 37-45% for those whose tumors had either *MYCN* amplification or segmental alterations (8). Similar results were seen in an independent study of 556 tumors – in which a higher overall number of chromosome breakpoints also was significantly associated with poor outcomes. Notably, in both studies those whose tumors have both segmental and numerical alterations behave as those with segmental defects – losing the apparent benefit of the numerical alterations. Similarly, with the exception of a novel chromosome 6q loss, the specific nature of the segmental alteration was less important than the presence of any segmental change. From this, it is evident that numerical genomic alterations are linked to a better prognosis, while on the contrary structural alterations are linked to poorer outcome and increased incidence of relapse (8, 56, 65). The mechanisms that lead to those states, however, is not clear. We propose an intermediate state where the tumor, or subclones within, become aneuploid due to the loss or gain of whole chromosomes. This state then predisposes the cells to acquire further genomic insults such as DNA damage, chromosome fragmentation, or chromothripsis, that result in segmental chromosome alterations.

## *USP24*: a CIN gene associated with poor outcomes in neuroblastoma

We performed an on online computational screen using the PREdiction of Clinical Outcomes from Genomic Profiles tool (PRECOG; https://precog.stanford.edu/) to identify novel therapeutic targets in NBL (66). This tool analyzes the expression of genes in correlation with survival statistics and calculates a z-score to represent its impact on outcomes. Scores near zero are predicted to have little impact on disease outcomes, whereas significantly low, or high, z-scores suggest that reduced expression or elevated expression respectively is associated with por outcomes. Our screen focussed on the gene family encoding de-ubiquitinating enzymes (DUBs) – as these are emerging as novel therapeutic targets. When examining the impact of 95 DUBs on outcomes in NBL, we found that reduced expression of *USP24* had the largest impact on outcomes, with a z-score of -10.14 (0.38 percentile) suggesting that *USP24* reductions have a powerful negative impact in this disease (67). For comparison, the canonical NBL oncogene *MYCN* has a z-score of +8.18 (97.34 percentile) - in line with the known relationship between high expression and aggressive disease. We further found that patients whose tumors had reduced expression of *USP24*, defined as less than the 20^th^ percentile, were significantly more likely to suffer disease progression and to die of any cause. *USP24* is encoded on chromosome 1p32.3 – and area of the genome that is frequently lost in neuroblastoma tumors (68, 69). It is notable then, that cells lacking USP24 have a significant increase in erroneous mitosis, with a particular increase in lagging chromosomes (67). These cells and tissues from mice missing even 1 copy of the *Usp24* gene have a significant increase in numerical chromosome aneuploidy. We identified that USP24 is required to preserve expression of CRMP2 – a microtubule assembly factor encoded by the *DPYSL2* gene. We found CRMP2 to co-localize with spindle microtubules. Cells lacking USP24 had a significant reduction in spindle associated CRMP2 – a defect that was corrected by proteasome inhibition. Furthermore, restoring the expression of CRMP2 was able to correct the mitotic defect seen in cells lacking USP24. Looking again at human NBL, we also found that low *USP24* was highly correlated with a high CIN25 score, supportive of the connection between *USP24* and CIN in these tumors. Importantly, the poor survival seen in patients with tumors with low levels of *USP24* is significantly improved by higher levels of CRMP2 (*DPYSL2*). Taken in aggregate, this data strongly implicates *USP24* as a novel tumor suppressor gene in NBL that, when lost, promotes CIN and disease aggression.

## Connecting the dots between numerical and segmental chromsome alterations

**Figure 2:**
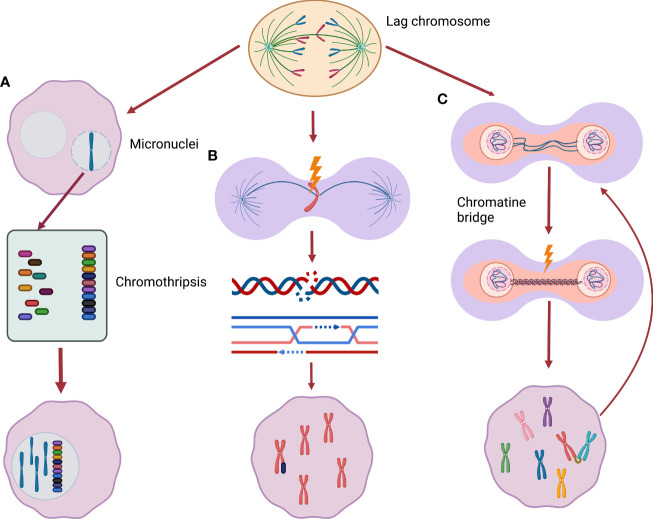
From whole chromosome mis-segregation to segmental alterations. **(A)** Lagging chromosomes may be incorporate into micronuclei, encountering chromothripsis (a phenomenon of massive chromatin fragmentation) during the following cell replication cycle. These severe rearranged chromosomes can be subsequently included back in the main nucleus. **(B)** Missegregated chromosomes can be frequently damaged during cytokinesis activating DNA double-strand break response leading to new segmental alterations. **(C)** Chromatin bridges can cause chromatin breakage triggering breakage-fusion-bridge cycle, a well described origin of segmental alterations.

The association of *USP24* reduction and aggressive NBL is seemingly at odds with the general association between numerical chromosome instability and favorable disease outcomes. However, the mutually exclusive nature of the literature that separates numerical from segmental chromosome alterations may be an oversimplification when it comes to the underlying biology. As will be discussed further below, recently studies have demonstrated that whole chromosome missegregation leads to the subsequent development of structural chromosome damage. We propose a model by which aggressive NBL with segmental chromosome alterations may arise from an intermediate stage characterized by whole chromosome aneuploidy (**Figure 2**).

As mentioned previously, the most common chromosome segregation error observed in cancer cells is chromosome lagging. This error, the result of mal-attachment of spindle microtubules to sister chromatid kinetochores, often results in genetic material separate from the main chromatin mass as cells enter interphase following cytokinesis (70). Consequentially as the nuclear membrane reforms, the erroneous segregation the lagged chromosomes can be surrounded by a distinct nuclear membrane forming a micronucleus. This phenomenon has led to micronuclei (MNi) being used as a marker of CIN. The Pellman laboratory observed that these MNi are highly susceptible to DNA damage in the next cell division cycle (71, 72). By following the fate of cultured cells with MNi, the group found that chromatin in MNi are subject to defective DNA replication during subsequent S-phases – with resulting DNA damage that may be catastrophic. Some cells encountered severe fragmentation of the chromatin within the MNi, a process known as chromothripsis. Moreover, the resulting fragmented chromatin was often re- incorporated into the main nuclear genome (71). In such way, the new rearrangements can be merged into the new daughter cells genome. The labs of Kops and Medema also demonstrated that missegregated chromosomes commonly develop DNA damage – with the subsequent double-strand break response leading to the development of chromosome translocations (73). While these studies were not performed specifically in NBL cells, it provides clear evidence that numerical chromosome missegregation may lead to severe DNA damage and subsequent structural chromosome rearrangements.

In addition to the consequences resulting from lagging chromosomes, another error that may produce segmental chromosome alterations is the chromatin bridge (74, 75). Chromatin bridges are thin chromatin strings that connect the separating chromosome masses during anaphase. In many instances, these bridges persist through telophase with chromosome breaks introduced during cytokinesis. Anaphase bridges often originate from incomplete DNA replication or fusion of telomeric end regions of two chromosomes which are later pulled to different poles during anaphase (dicentrinc chromosomes). Some bridges, in particular ultrafine DNA bridges can resolve without permanent DNA damage (75), others can lead to chromatin breakage causing DNA damage (73). Chromosomes harboring chromatin bridges often develop a variety of structural rearrangements (76) and often break into multiple fragments (74, 77). These breaks may trigger chromosome breakage-fusion-bridge (BFB) cycles – a known contributor to chromosomal structural rearrangements (76). Notably, recent evidence suggests that – as with lagging chromosomes – the attempted resolution of dicentric-chromosome induced chromatin bridges may result in severe chromosome fragmentation – including chromothripsis.

## Discussion

CIN, aneuploidy, and segmental chromosome alterations are a pan-cancer feature. The apparent impact of these changes on prognosis – differs widely across tumor types – and in neuroblastoma numerical changes are often associated with good outcomes (Bilke S et al., 2005; Janoueix-Lerosey I et al., 2009; Fusco P et al., 2018). Here we review evidence that aneuploidy can be a first step towards the development of segmental alterations and propose a model in which segmental alterations in neuroblastoma may arise through this intermediate step of numerical aneuploidy. As summarized above there are several scientific studies that demonstrate an association between these two phenomena. The mechanisms that may connect whole-chromosome missegregation, aneuploidy, and the subsequent development of segmental chromosome alterations provide a potential explanation for our observation that the loss of USP24 may promote aggressive NBL. We have found that reduced expression of USP24 leads to abnormalities in the mitotic spindle and a significant increase in chromosome missegregation and aneuploidy in a mouse model. Surprisingly, there are no data involving the intercross of aneuploidy mouse models with those predisposed to neuroblastoma. Our *Usp24* deficient mouse model may be a useful tool to help further explore this question. Such experimental models may shed light on the role of aneuploidy in initial tumor development, tumor progression, tumor evolution and of course in therapy responses. As there are hundreds of genes required for accurate cell division – it is unclear whether any cause of CIN may contribute to neuroblastoma development – or whether cell, tissue, or developmentally specific gene expression influences how the alteration of any one CIN gene impacts downstream events. We predict that specific mechanisms that produce CIN in this context will have important prognostic effects – since disease outcomes vary more by the presence or absence of segmental anomalies rather than the presence of any selected event.

## Author contributions

LP, SH, and PG co-authored, edited, and approved the manuscript. All authors contributed to the article and approved the submitted version.

## Acknowledgments

Figures were created with Biorender.com. The work was supported by grants to P.J. Galardy including from the Howard Hughes Medical Institute (HHMI), Fraternal Order of Eagles, and a Mayo Clinic Career Development award.

## Conflict of interest

The authors declare that the research was conducted in the absence of any commercial or financial relationships that could be construed as a potential conflict of interest.

## Publisher’s note

All claims expressed in this article are solely those of the authors and do not necessarily represent those of their affiliated organizations, or those of the publisher, the editors and the reviewers. Any product that may be evaluated in this article, or claim that may be made by its manufacturer, is not guaranteed or endorsed by the publisher.
